# Controversies in the Management of the First Time Shoulder Dislocation

**DOI:** 10.2174/1874325001711011001

**Published:** 2017-08-31

**Authors:** José Luis Avila Lafuente, Santos Moros Marco, José Manuel García Pequerul

**Affiliations:** Department of Orthopedic Surgery, Hospital MAZ, Zaragoza, Spain

**Keywords:** Shoulder dislocation, Instability, Treatment, Arthroscopy, First dislocation, Review

## Abstract

**Background:**

Traditionally, initial management of first anterior shoulder dislocations consists of reduction of the glenohumeral joint followed by a period of immobilization and subsequent physical therapy to recover shoulder range of motion and strength. This traditional approach in management is now controversial due to the high rate of recurrence. The aim of this paper is to review and discuss the literature about the global management of patients presenting with first-time traumatic anterior glenohumeral dislocation, analyzing the factors that affect shoulder instability after the first episode of dislocation.

**Methods:**

Scientific publications about the management of first-time shoulder dislocations are reviewed. Pubmed is used for that and no limit in the year of publication are stablished. These papers and their conclusions are discussed.

**Results:**

Younger patients, patient´s activities and the kind of injury are the most important factors related to the shoulder instability after a first time traumatic dislocation. Authors that recommend surgical treatment after the first episode of dislocation argue that the possibilities of recurrence are high and therefore surgery should be performed before its occurrence. Other authors, however, argue that surgical treatment is demanding, and keep in mind that complications, such as recurrence, stiffness and pain after surgery, are still present.

**Conclusion:**

Currently, there is still no consensus in the literature with regard to the management of first episode of shoulder dislocation. It is necessary to analyze carefully every individual case to manage them more or less aggressive to obtain the best result in our practice.

## 1 INTRODUCTION

Shoulder dislocation is the most frequent large joint dislocation in the human body, with an incidence of 1,7% (8, 2-17 cases per 100000 people per year) [1]. The main cause (95%) of the first time dislocation is a traumatic event, and shoulder dislocations account for approximately 50% of all joint dislocations presenting to emergency departments [2].

Traditionally, the initial management of a patient with a first episode of anterior shoulder dislocation is the reduction of the glenohumeral joint, followed by immobilization in a “safe position” for three weeks. This is followed by a period of physical therapy in order to recover shoulder range of motion and strength.

This traditional management has been put into question due to the high risk of recurrence, especially in the younger population. Alternatives to these initial protocols, including the possibility of early surgical stabilization, have been proposed and will be discussed in this review.

## 2 ACUTE MANAGEMENT OF THE PATIENT WITH A FIRST EPISODE OF ANTERIOR SHOULDER DISLOCATION

The key to a successful reduction of a first-time shoulder dislocation is to get adequate muscular relaxation. Experienced clinicians are usually able to get a successful reduction without any analgesia or sedation. In these cases, reduction techniques as the Milch technique or scapular manipulation, in which minimal traction is applied, should be used. If these initial attempts are unsuccessful, the clinician could then proceed to use intra-articular analgesia (usually 10 ml of 1% lidocaine infiltrated inside the joint through a lateral approach), as it has been shown to be safe and effective. Alternatively, sedation (intravenous benzodiazepines and/or nitrous oxide and oxygen) with or without analgesia (major opioids) could be used [3].

After a successful reduction the traditional management consisted in arm immobilization in a sling with the shoulder in an adduction and internal rotation for 3 weeks. Nowadays, both the duration of immobilization and the arm position are controversial issues.

The classical study by Kiviluoto *et al*. in 1980, included patients aged less than 30 years with primary shoulder dislocations. The authors found that the recurrence rate was 50% in the 26 patients who were immobilized for one week, while it was 22% in the 27 patients who were immobilized for three weeks [4]. Despite these findings, more recent evidence suggests that recurrent instability is not affected by the duration of the immobilization in internal rotation [5].

Itoi *et al*.´s study [6] which involved a total of 198 patients immobilized for three weeks in either internal rotation (IR) or 10º of external rotation (ER), found a significantly lower recurrence rate in the ER immobilization group compared to the IR group (26% *vs*. 42%). They also noted that immobilization in the ER position was especially beneficial for patients aged 30 years or younger. Different cadaveric and MRI studies have shown that ER immobilization after reduction might provide improved healing of the labral tears derived from the dislocation. On the other side, a large randomized controlled trials found that external rotation position after reduction did not decrease the rate of recurrence [7]. These reports and others were evaluated in a Cochrane systematic review that concluded that there was not enough evidence to recommend a specific immobilization protocol [8].

Irrespective of the choice of position and immobilization time, this traditional approach in the management of glenohumeral dislocations is now controversial due to the high rate of recurrence observed, with rates as high as 92-96% in young active patients [9].

The three major reasons cited in the literature for supporting immediate stabilization over conservative treatment are: (a) there is an unacceptable high risk of recurrence in the young athletic population; (b) that recurrent instability causes significant and progressive soft tissue and bony damage; and (c) there is a clear improvement in the quality of life conferred by surgery [10].

Hoveluis and Saeboe [11] followed 223 patients with first-time shoulder dislocations prospectively for up to 25 years and found significant shoulder arthropathy 25 years after the first episode. Arthropathy was more prevalent in patients presenting several dislocations (40%) compared with those patients that just suffered one episode (18%). Buscayret *et al*. [12] reported similar findings in their study evaluating osteoarthritis after anterior shoulder dislocation. They also reported that, as the number of instability episodes increased, the rate of postoperative osteoarthritis also increased.

Several recent studies have shown a decrease in recurrence of dislocation if early surgical intervention is performed by arthroscopic stabilization when comparing it to non-operative treatment [13, 14].

## 3 FACTORS THAT AFFECT OUTCOME AFTER AN INITIAL EPISODE OF ANTERIOR SHOULDER DISLOCATION

There are many aspects like age, activity, lesions associated in the shoulder and more, to evaluate in our patients before to decide the adequate management of the primary shoulder dislocation. The purpose of this review was to analyze the factors that affect to shoulder instability after the first time dislocation to help us in managing more or less aggressive each particular case, to obtain the best result in our practice.

### 3.1 Age

The risk of recurrent instability is significantly associated with the age at the time of initial dislocation [15]. Gumina and Postacchini [16] reviewed traumatic fist-time anterior dislocation in patients 60 years or over and found a recurrence rate of 22%. On the other side of the spectrum, Marans *et al*. [17] looked at children with open physis and found a 100% recurrence rate. Hovelius *et al*. [18] showed the results of a study with a 25-year follow-up on conservative treatment for anterior shoulder dislocations. They found recurrences in 72% of patients aged 12-22 years, 56% of patients aged 23-29 years and 27% of those older than 30 years. The fact that 20% of patients aged 12-22 years with recurrent instability are still stable 15-25 years after first injury has to be taken into account.

The high incidence of recurrent dislocation of the glenohumeral joint among adolescence may be partially explained by the profile of existing collagen presented in the capsule and other tissues of the shoulder in this population. The greatest quantity of collagen type 3 with elastic properties in tendons and ligaments may help explain the higher tendency of younger patients to recurrent dislocation of the shoulder compared with older patients [19].

In Boone *et al*.´s literature review on the pathoanatomy of first-time anterior dislocation, early surgical repair was advocated in young patients less than 25 years old to improve the quality of life and outcomes and to decrease the incidence of recurrent dislocation and associated arthropathy [20]. Treatment should be based on the method that results in the best outcome but they recommended overall arthroscopic suture anchors repair.

### 3.2 Pathology of Acute Dislocation

Anterior glenohumeral dislocations frequently take place with the arm in abduction and external rotation, leading to predictable patterns of injury to the labrum, capsuloligamentous structures, glenoid and humeral head.

After a first-time shoulder dislocation, a combination of lesions can originate a chronic instability, mainly, those injuries involving the inferior glenohumeral ligament, that is the most important passive stabilizer of the shoulder restricting the anterior humeral translation in abducted shoulder [21].

The stability of the glenohumeral joint is guaranteed by the glenoid labrum. This structure creates a socket-deepening effect in order to prevent shoulder dislocation. The anteroinferior labrum also serves as the anchor point for the inferior glenohumeral ligament [22].

**Fig. (1):**
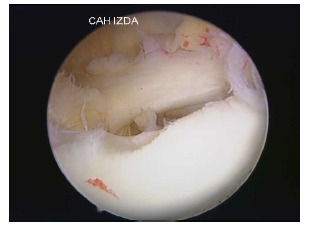
Arthroscopic view of a chronic Bankart lesion with affected capsular tissue.

**Fig. (2):**
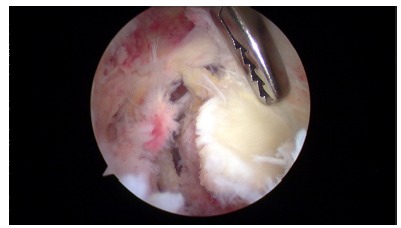
Arthroscopic view of a small bony Bankart lesion.

An avulsion of the anteroinferior labrum from the glenoid rim is known as a Bankart lesion [23] which has been regarded as the essential lesion in such cases [24]. The high recurrence rate observed with the use of conservative treatment could be attributed to a labrum that heals in a non anatomic position [25] (Fig. **1**). Other lesions that may be associated with traumatic anterior dislocations include humeral head Hill-Sachs lesions, SLAP tears (superior labrum tear from anterior to posterior), capsuloligamentous tears, rotator cuff tears and glenoid rim fractures [20] (Fig. **2**).

Baker *et al*. [26] performed an arthroscopic evaluation of 45 patients who had suffered an acute anterior shoulder dislocation in order to identify and classify the intraarticular injuries that might predict recurrent dislocations. Six shoulders (group 1) had capsular tears with no labral lesions. These shoulders were not unstable under anesthesia and had no or minimal hemarthrosis. Eleven shoulders (group 2) presented capsular tears and partial labral detachments. These shoulders were mildly unstable and had mild to moderate hemarthrosis. And finally 28 shoulders (group 3) presented capsular tears and labrum detachments. These shoulders were grossly unstable with large hemarthrosis. In this group 3, a complete capsular/ labral detachment was found and usually a hill-Sachs lesion was associated.

In a study evaluating fist-time traumatic dislocations in young patients, Taylor and Arciero [27] documented that 97% of their patients have Bankart lesions with no clear evidence of capsular injury. They also noted that 89% of their patients presented a Hill-Sach lesion, although these were small and did not seem to significantly affect stability on arthroscopic evaluation.

A study which examined the bony lesions of 570 patients who underwent a stabilizing procedure, found that the presence of bony glenoid rim injuries was a risk factor in the development of preoperative osteoarthritis [12]. Griffith *et al*. [28] assessed the relationship between the frequency of dislocation and the prevalence, pattern and spectrum of glenoid bone loss in anterior glenohumeral dislocation, using computed tomography. They reported that glenoid bone loss was found in 41% of their patients with first-time dislocation and in 86% of the patients with recurrent dislocation, with moderate correlation between the number of dislocations and the severity of the glenoid bone loss.

Burkhart and De Beer [29] were one of the first to illustrate the major risk of redislocations, up to 67%, associated with glenoid erosion. But on the other side, Salomonsson *et al*. [30] published that in patients above 30 years old, the presence of greater tuberosity fractures and fractures of the glenoid rim could be considered as a good predictor of stability and function of the shoulder after a traumatic dislocation of the glenohumeral joint.

**Fig. (3):**
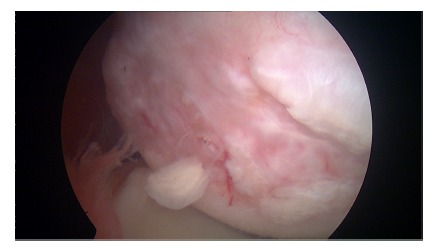
Arthroscopic view of a chronic large humeral Hill-Sachs lesion.

**Fig. (4):**
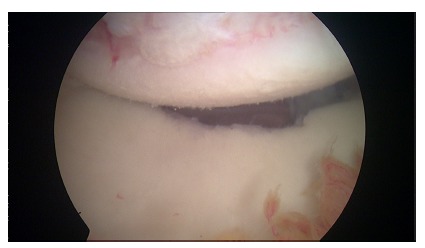
Arthroscopic view of a enganging Hill-Sachs lesion.

The patients with large humeral Hill-Sachs lesions have enough apprehension with the arm in abduction and external rotation that they voluntarily curtail overhead activities (Fig. **3**). Burkhart *et al*. [31] observed arthroscopically that with the arm abducted to 90º, if the shoulder was externally rotated over 30º, the Hill-Sachs lesion could engage the anterior corner of the glenoid, and the patient would sense that engagement as a popping or catching sensation considered as instability (Fig. **4**). Burkhart *et al*. [29] defined the humeral engaging Hill-Sachs lesion as the one that presents the long axis of its defect parallel to the anterior glenoid with the shoulder in a functional position of abduction and external rotation. These authors also defined the inverted-pear configuration caused by a large bony Bankart or a Bankart lesion without an associated bony fragment but with a significant compression defect, as a shape of the glenoid where the top half of the glenoid is wider than the lower half. They showed in their study the association of the inverted-pear configuration with recurrent dislocation.

### 3.3 Patient´s Activity

In athletes and heavy manual workers, mainly if they perform overhead activities, the case could be made for early surgery after a first-time dislocations to improve their outcomes.

Younger patients and practitioners of high-impact sports are more prone to recurrence after an initial shoulder dislocation. However, just about 50% of these will require surgery [32].

Thus, it would be helpful to use specific tools to predict the risk of recurrence after the first traumatic episode of glenohumeral dislocation. These tools would help physicians to identify patients at high risk of redislocation and submit them to a primary surgery in order to reduce the risk of recurrence.

## 4 THE CASE FOR EARLY SURGICAL TREATMENT

Physicians that recommend surgical treatment after the first episode of dislocation argue that the possibilities of recurrence are high and therefore surgery should be performed before its occurrence. Other authors, however, argue that surgical treatment is demanding, and keep in mind that complications, such as recurrence, stiffness and pain after surgery, are still present [33, 34].

**Fig. (5):**
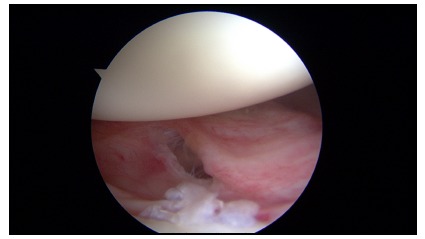
Arthroscopic view of an acute glenohumeral capsular tear after first-time shoulder dislocation.

**Fig. (6):**
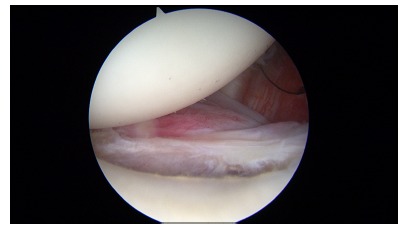
Arthroscopic view of an acute Bankart lesion after first-time shoulder dislocation.

After the first episode of shoulder dislocation, the patients do not usually present extensive intra-articular tears, however, they usually have intact secondary stabilizers and good potential for healing of the damaged soft tissues is present. Based on these findings, non- operative management can be right choice for first-time dislocations. On the other side the pathology worsens without surgical intervention. Single dislocations are typically associated with acute pathology and these patients are good candidates for successful arthroscopic repair (Figs. **5** and **6**). Sachs *et al*. [32] aimed to identify patients at high risk for shoulder re-dislocation and determine whether these high-risk patients were best served by immediate surgical stabilization by prospectively following up on 131 patients for five years. They found that younger patients who were involved in contact or collision sports, or who required overhead use of the arm, were more likely to have re-dislocation of the shoulder than their less active peers, or older persons. However, even in the highest-risk group, only approximately 50% of the patients with shoulder re-dislocations requested surgical treatment within the follow-up period. Thus, the authors could not justify early surgical treatment based on the presumption of future dislocations, unhappiness and disability.

Few randomized clinical trials have been performed in order to determine the best treatment option after the first episode of traumatic dislocation of the shoulder. Bottoni *et al*. in 2002 [34] with 24 patients less than 26 y.o., published 75% recurrence rate in non-surgical management and 11% in early surgical repair. Kirkley *et al*. in 2005 [13] studied 40 patients less than 30 y.o. and noted 47% and 16% respectively and finally Jakobsen *et al*. in 2007 [14] controlled 76 patients aged between 15 and 39 years, and observed 54% recurrence rate in nonsurgical group and 3% in the surgical group.

**Fig. (7):**
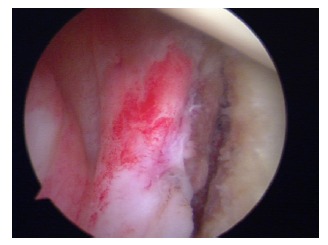
Arthroscopic view of an acute bony Bankart lesion previous to the repair.

**Fig. (8):**
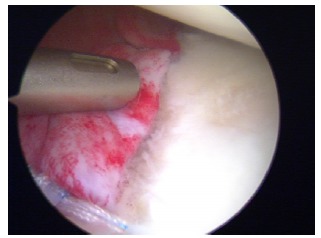
Arthroscopic view of an acute bony Bankart lesion after the repair by a Sugaya technique.

**Fig. (9):**
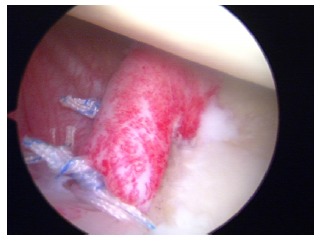
Arthroscopic view of an acute bony Bankart lesion at the end of the previous Sugaya procedure.

When a bony bankart lesion is identified after the first episode of dislocation, the case can be made for early repair of the bony lesion. Both Porcellini *et al*. in 2002 [35] and Sugaya *et al*. in 2005 [36] described their surgical techniques using suture anchors under all-arthroscopic control in a series of bony Bankart lesion within 3 months from the initial trauma and affecting less than 25% of the glenoid surface, to avoid permanent instability, with good results (Figs. **7** - **9**).

No studies have demonstrated that surgical treatment performed after a primary dislocation means better outcomes than that performed after a recurrence but Boone *et al*. [20] recently published a review of the literature about the management of first episode of shoulder dislocations and they concluded that arthroscopic surgical repair using suture anchors especially in the at-risk group less than 25 years of age is a viable option that challenges the traditional standard approach of immobilization as the definitive treatment.

Habermeyer *et al*. [37] studied a spectrum of patients with traumatic anterior glenohumeral instability, ranging from first-time dislocators to chronic subluxers, and revealed a progressive labral-ligamentous injury and degeneration with increasing episodes of dislocation. The authors described four phases in the development of injury in which there is a sequential loss of the anterior “hinges” (labrum and inferior glenohumeral ligament) and then irreversible plastic deformation and degeneration of the anterior structures occurring by the third dislocation (which they named the “point of no return”). Wasserstein *et al*. [38] studied a large population (5900 shoulder stabilizations performed between 2003 and 2008) and found that the most important risks factors for recurrence were 1) three or more recurrent dislocations prior to operative stabilization, and 2) age less than 20 years.

Many studies have evaluated surgical procedures that can be undertaken after an acute traumatic shoulder dislocation. A study by Wintzell *et al*. [39] which aimed to evaluate the effect of arthroscopic lavage as a form of treatment for acute anterior glenohumeral dislocation, reported a recurrence rate of 43% in patients who received nonsurgical treatment, while it was only 13% in patients who received an arthroscopic lavage. Robinson *et al*. [40] found that arthroscopic repair of a Bankart lesion after first time traumatic anterior glenohumeral dislocation reduced the risk of recurrence by 76%; they also found that the risk of all recurrent instability was reduced by 82% in the group of patients that underwent Bankart repair, compared to the group that underwent arthroscopy and lavage alone. They concluded that marked treatment benefit could be derived from primary arthroscopic repair of a Bankart lesion, different from the so-called background therapeutic effect of arthroscopic examination and lavage of the joint. However they noted that primary repair did not seem to cause a functional benefit to patients with a stable shoulder at two years after the dislocation.

A systematic review showed that there is limited available evidence supporting primary surgery for young adults and athletes, frequently male, and practitioners of highly demanding physical activities after the first traumatic dislocation [41].

## 5 AUTHOR´ PREFERED MANAGEMENT OF THE PATIENT WITH A FIRST-TIME DISLOCATION

In our institution (a hospital that focuses in worker´s compensation cases and some professional sportsmen) we only operate first time shoulder dislocations associated with glenoid fractures with more than 10-15% of bone loss in patients less than 45 years old. In this case, we perform an arthroscopic Sugaya technique [36], if we treat a small bony fragment or screw fixation for bigger fragments. We also operate early on patients with irreductible dislocations and the initial permanent instable cases.

The majority of patients who suffer a traumatic first-time shoulder dislocation without bony Bankart are thus not operated initially in our institution. We treat them conservatively by immobilization with a sling in adduction and internal rotation for 20 days. In this period isometric exercises without causing pain and passive and assisted elevation up to 100º are allowed. After this initial phase, a non- aggressive rehabilitation protocol is performed until full recovery, usually during two months. We focus in an extended follow-up in younger patients (less than 30 years old). Based on the studies of Habermeyer *et al*. [37], Wasserstein *et al*. [38] and others, we perform a surgical repair always after the second episode of shoulder dislocation in patients less than 30 years old and frequently also in those patients less than 40 years old.

## CONCLUSION

Currently, there is still no consensus in the literature with regard to the global management of patients presenting with first-time traumatic anterior dislocation of the shoulder. The traditional standard approach of immobilization as a definitive treatment after the reduction is being challenged due to the high rate of recurrence, but it is not clearly defined how to immobilize and for how long.

Recurrent instability has been the only outcome measure for many years. However other facts such as continued apprehension, disability when returning to work or sports, established the quality of life outcomes measures and the appearance of post-traumatic osteoarthritis are critical in evaluating the treatment outcomes.

In our opinion, in young patients less than 20-25 years old, after fist-time shoulder dislocation, it is necessary to evaluate carefully every individual case, to decide if an early surgical repair is necessary. The wide majority of our patients are treated conservatively after first time dislocation. However, we keep in mind the presence of pain, loss of articular range and clear apprehension in daily activities in the examination. We also consider if the affected shoulder is the dominant side and the professional and/or sportive activities that the patient use to perform. And above all, in these young patients we also investigate the presence of glenoid bone loss, big size Hill-Sachs lesions and large inferior soft tissue tears, to indicate or not early surgery.

## References

[1] Krøner K., Lind T., Jensen J. (1989). The epidemiology of shoulder dislocations.. Arch. Orthop. Trauma Surg..

[2] Blake R., Hoffman J. (1999). Emergency department evaluation and treatment of the shoulder and humerus.. Emerg. Med. Clin. North Am..

[3] Dala-Ali B., Penna M., McConnell J., Vanhegan I., Cobiella C. (2014). Management of acute anterior shoulder dislocation.. Br. J. Sports Med..

[4] Kiviluoto O., Pasila M., Jaroma H., Sundholm A. (1980). Immobilization after primary dislocation of the shoulder.. Acta Orthop. Scand..

[5] Kuhn J.E. (2006). Treating the initial anterior shoulder dislocationan evidence-based medicine approach.. Sports Med. Arthrosc. Rev..

[6] Itoi E., Hatakeyama Y., Sato T., Kido T., Minagawa H., Yamamoto N., Wakabayashi I., Nozaka K. (2007). Immobilization in external rotation after shoulder dislocation reduces the risk of recurrence. A randomized controlled trial.. J. Bone Joint Surg. Am..

[7] Liavaag S., Brox J.I., Pripp A.H., Enger M., Soldal L.A., Svenningsen S. (2011). Immobilization in external rotation after primary shoulder dislocation did not reduce the risk of recurrence: a randomized controlled trial.. J. Bone Joint Surg. Am..

[8] Handoll H.H., Hanchard N.C., Goodchild L., Feary J. (2006). Conservative management following closed reduction of traumatic anterior dislocation of the shoulder.. Cochrane Database Syst. Rev..

[9] te Slaa R.L., Brand R., Marti R.K. (2003). A prospective arthroscopic study of acute first-time anterior shoulder dislocation in the young: a five-year follow-up study.. J. Shoulder Elbow Surg..

[10] Baumgarten K.M., Wright R.W. (2007). Ease of tying arthroscopic knots.. J. Shoulder Elbow Surg..

[11] Hovelius L., Saeboe M. (2009). Neer Award 2008: Arthropathy after primary anterior shoulder dislocation223 shoulders prospectively followed up for twenty-five years.. J. Shoulder Elbow Surg..

[12] Buscayret F., Edwards T.B., Szabo I., Adeleine P., Coudane H., Walch G. (2004). Glenohumeral arthrosis in anterior instability before and after surgical treatment: incidence and contributing factors.. Am. J. Sports Med..

[13] Kirkley A., Werstine R., Ratjek A., Griffin S. (2005). Prospective randomized clinical trial comparing the effectiveness of immediate arthroscopic stabilization versus immobilization and rehabilitation in first traumatic anterior dislocations of the shoulder: long-term evaluation.. Arthroscopy.

[14] Jakobsen B.W., Johannsen H.V., Suder P., Søjbjerg J.O. (2007). Primary repair versus conservative treatment of first-time traumatic anterior dislocation of the shoulder: a randomized study with 10-year follow-up.. Arthroscopy.

[15] Robinson C.M., Howes J., Murdoch H., Will E., Graham C. (2006). Functional outcome and risk of recurrent instability after primary traumatic anterior shoulder dislocation in young patients.. J. Bone Joint Surg. Am..

[16] Gumina S., Postacchini F. (1997). Anterior dislocation of the shoulder in elderly patients.. J. Bone Joint Surg. Br..

[17] Marans H.J., Angel K.R., Schemitsch E.H., Wedge J.H. (1992). The fate of traumatic anterior dislocation of the shoulder in children.. J. Bone Joint Surg. Am..

[18] Hovelius L., Olofsson A., Sandström B., Augustini B.G., Krantz L., Fredin H., Tillander B., Skoglund U., Salomonsson B., Nowak J., Sennerby U. (2008). Nonoperative treatment of primary anterior shoulder dislocation in patients forty years of age and younger. a prospective twenty-five-year follow-up.. J. Bone Joint Surg. Am..

[19] Hayes K., Callanan M., Walton J., Paxinos A., Murrell G.A. (2002). Shoulder instability: management and rehabilitation.. J. Orthop. Sports Phys. Ther..

[20] Boone J.L., Arciero R.A. (2010). First-time anterior shoulder dislocations: has the standard changed?. Br. J. Sports Med..

[21] Bergin D. (2009). Imaging shoulder instability in the athlete.. Magn. Reson. Imaging Clin. N. Am..

[22] Turkel S.J., Panio M.W., Marshall J.L., Girgis F.G. (1981). Stabilizing mechanisms preventing anterior dislocation of the glenohumeral joint.. J. Bone Joint Surg. Am..

[23] Rowe C.R., Patel D., Southmayd W.W. (1978). The Bankart procedure: a long-term end-result study.. J. Bone Joint Surg. Am..

[24] Robinson C.M., Dobson R.J. (2004). Anterior instability of the shoulder after trauma.. J. Bone Joint Surg. Br..

[25] Kirkley A., Griffin S., Richards C., Miniaci A., Mohtadi N. (1999). Prospective randomized clinical trial comparing the effectiveness of immediate arthroscopic stabilization *versus* immobilization and rehabilitation in first traumatic anterior dislocations of the shoulder.. Arthroscopy.

[26] Baker C.L., Uribe J.W., Whitman C. (1990). Arthroscopic evaluation of acute initial anterior shoulder dislocations.. Am. J. Sports Med..

[27] Taylor D.C., Arciero R.A. (1997). Pathologic changes associated with shoulder dislocations. Arthroscopic and physical examination findings in first-time, traumatic anterior dislocations.. Am. J. Sports Med..

[28] Griffith J.F., Antonio G.E., Yung P.S., Wong E.M., Yu A.B., Ahuja A.T., Chan K.M. (2008). Prevalence, pattern, and spectrum of glenoid bone loss in anterior shoulder dislocation: CT analysis of 218 patients.. AJR Am. J. Roentgenol..

[29] Burkhart S.S., De Beer J.F. (2000). Traumatic glenohumeral bone defects and their relationship to failure of arthroscopic Bankart repairs: Significance of the inverted-pear glenoid and the humeral engaging Hill-Sachs lesion.. Arthroscopy.

[30] Salomonsson B., von Heine A., Dahlborn M., Abbaszadegan H., Ahlström S., Dalén N., Lillkrona U. (2010). Bony Bankart is a positive predictive factor after primary shoulder dislocation.. Knee Surg. Sports Traumatol. Arthrosc..

[31] Burkhart S.S., Danaceau S.M. (2000). Articular arc length mismatch as a cause of failed bankart repair.. Arthroscopy.

[32] Sachs R.A., Lin D., Stone M.L., Paxton E., Kuney M. (2007). Can the need for future surgery for acute traumatic anterior shoulder dislocation be predicted?. J. Bone Joint Surg. Am..

[33] Simonet W.T., Melton L.J., Cofield R.H., Ilstrup D.M. (1984). Incidence of anterior shoulder dislocation in Olmsted County, Minnesota.. Clin. Orthop. Relat. Res..

[34] Bottoni C.R., Wilckens J.H., DeBerardino T.M., DAlleyrand J.C., Rooney R.C., Harpstrite J.K., Arciero R.A. (2002). A prospective, randomized evaluation of arthroscopic stabilization versus nonoperative treatment in patients with acute, traumatic, first-time shoulder dislocations.. Am. J. Sports Med..

[35] Porcellini G., Campi F., Paladini P. (2002). Arthroscopic approach to acute bony Bankart lesion.. Arthroscopy.

[36] Sugaya H, Kon Y, Tsuchiya A (2005). Arthroscopic repair of glenoid fractures using suture anchors.. Arthroscopy.

[37] Habermeyer P., Gleyze P., Rickert M. (1999). Evolution of lesions of the labrum-ligament complex in posttraumatic anterior shoulder instability: a prospective study.. J. Shoulder Elbow Surg..

[38] Wasserstein D., Dwyer T., Veillette C., Gandhi R., Chahal J., Mahomed N., Ogilvie-Harris D. (2013). Predictors of dislocation and revision after shoulder stabilization in Ontario, Canada, from 2003 to 2008.. Am. J. Sports Med..

[39] Wintzell G., Haglund-Akerlind Y., Ekelund A., Sandström B., Hovelius L., Larsson S. (1999). Arthroscopic lavage reduced the recurrence rate following primary anterior shoulder dislocation. A randomised multicentre study with 1-year follow-up.. Knee Surg. Sports Traumatol. Arthrosc..

[40] Robinson C.M., Jenkins P.J., White T.O., Ker A., Will E. (2008). Primary arthroscopic stabilization for a first-time anterior dislocation of the shoulder. A randomized, double-blind trial.. J. Bone Joint Surg. Am..

[41] Handoll H.H., Almaiyah M.A., Rangan A. (2004). Surgical versus non-surgical treatment for acute anterior shoulder dislocation.. Cochrane Database Syst. Rev..

